# Sustainable, Low Flammability, Mechanically-Strong Poly(vinyl alcohol) Aerogels

**DOI:** 10.3390/polym10101102

**Published:** 2018-10-05

**Authors:** Zhihan Cheng, Kimberly DeGracia, David A. Schiraldi

**Affiliations:** Department of Macromolecular Science & Engineering, Case Western Reserve University, Cleveland, OH 44106-7202, USA; kcd21@case.edu

**Keywords:** aerogels, poly(vinyl alcohol), flammability, tannic acid

## Abstract

Poly(vinyl alcohol) (PVA), tannic acid (TA) and sodium hydroxide (NaOH) were used to prepare low-flammability, mechanically-strong aerogels via an environmentally-friendly freeze-drying method. Because of the strong interaction between TA and PVA through hydrogen bonds, PVA/TA/NaOH aerogels exhibited compressive moduli as high as 12.7 MPa, 20 times that of the control PVA aerogel. The microstructure of the aerogels in this study showed that the addition of NaOH disrupted the typical “card of house” aerogel structure, while the samples with TA showed a stereoscopic uniform structure. The thermal stabilities of aerogels were tested by thermogravimetric analysis, showing both a decrease on the onset of decomposition temperature, and a reduction in decomposition rate after initial char formation. The peak heat release rate and total heat release, as measured by cone calorimetry, dropped by 69% and 54%, respectively, after adding TA and NaOH.

## 1. Introduction

Aerogels were first described by Kistler in 1931 utilizing a novel sol-gel and drying process [1]. This family of materials typically exhibit bulk densities ranging from 0.005 to 0.1 g/cm^3^ [2,3,4], and have attracted a great deal of attention because of their low densities, high specific surface areas, high porosities (and abilities to serve as absorbents) and low thermal conductivities. In thermal insulation, liquid absorbents, energy storage, catalyst supports, and supercapacitors have all been reported [5,6,7,8,9,10]. Beyond the use of silica, aerogels have also been demonstrated based upon clay, graphene, carbon nanotubes and polymers [6,11,12,13]. Poly(vinyl alcohol) (PVA), as a hydrophilic and water soluble polymer, is an ideal candidate for fabricating polymer aerogels because of its low toxicity, good chemical stability, and favorable mechanical properties [14]. Such PVA aerogels can be prepared using a sustainable, freeze-drying method [15]. Different methods have been reported with which to enhance the properties of PVA aerogels, including multiple freeze−thaw processes [16], radiation treatment [17,18,19], and addition of crosslinking agents [20] or incorporation of cellulose nanofibrils into the polymer [21]. Enhancements of mechanical properties have been demonstrated using the above methods, but few of these reduce the flammability of the aerogels, which are otherwise comparable to polymer foams. High flame retardancy PVA aerogels are ideal candidates to replace foams in the area of building materials and consumer products [16]. Incorporation of flame retardants, such as ammonium polyphosphate, potassium carbonate and silica gel reduced PVA aerogel flammability but at the cost of reducing mechanical properties [22,23].

**Figure 1:**
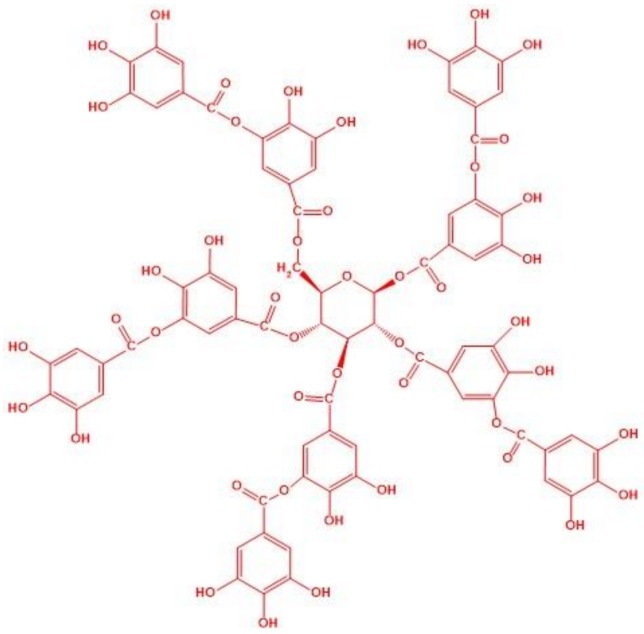
The structure of tannic acid.

Tannic acid (TA) is a naturally-occurring substance, which serves as a flame retardant when concentrated in the bark of trees, providing thermal and microbial protection [24,25]. TA (Figure 1) contains a central core of polyhydric alcohols connected to gallic acid through ester linkages. This structure lends itself to decomposition into graphite, making it a potential char-forming additive [26]. TA is considered to be sustainable and nontoxic, as opposed to traditional phosphorous–or halogen -based flame retardants, such as organophosphates, polybrominated biphenyls (PBBs) and polybrominated diphenyl ethers (PBDEs) [27]. TA can hydrogen bond with PVA through its many phenolic groups, potentially increasing the polymer aerogel’s mechanical properties. In order to provide an effective char layer when added to PVA, the concentration of TA cannot be too low (TA:PVA > 20%); when the concentration of PVA exceeds ratio over the limit (20%), a coagulation can occur in PVA solutions which hinders aerogel production [28,29]. Sodium hydroxide can be added to the polymer/TA mixtures to adjust pH and increase the solubility of the desired component [30,31], NaOH also has the function of catalyzing char formed from TA, because sodium cations could catalyze the dehydration of matrix, which contributes to the char layer [32]. To the best of our knowledge, there is no similar system reported in the literature. In the present work, TA and NaOH were incorporated into the PVA aerogel system to provide flame retardancy and improve mechanical properties. The resultant mechanical properties, morphologies, thermal stabilities, and combustion behaviors were investigated.

## 2. Experimental Section

### 2.1. Materials

Poly(vinyl alcohol) (PVA; M_w_ 13,000–23,000; 98% hydrolyzed), tannic acid (Sigma-Aldrich, St. Louis, MO, USA) and sodium hydroxide (pellets) (Aldon Corp, Rochester, NY, USA) were used without further purification. Deionized (DI) water was obtained using a Barnstead RoPure low-pressure, reverse-osmosis system (Lake Balboa, CA, USA).

### 2.2. Preparation of Aerogels

PVA solutions were prepared by stirring PVA powder with DI water for six hours at 90 °C. Sodium hydroxide and TA were mixed together with DI water under magnetic stirring for 30 min before addition into the PVA solutions. Compositions without tannic acid were prepared in the same manner as the aerogels containing TA; the solutions were mixed to the desired PVA concentrations at 50 °C until they were homogeneous suspensions. The suspensions were poured into 12.5 ml polystyrene vials (for compression testing) or cast into a 100 mm × 100 mm × 10 mm rectangular mold (for cone calorimetry testing) and frozen in a solid carbon dioxide/ethanol bath (–70 °C). These samples were freeze dried using a VirTis Advantage EL-85 lyophilizer, with the shelf temperature set to 25 °C, and the pressure set to under 10 μbar. The products are referred to as Px/Ty/S, where P represents PVA, T represents tannic acid and S represents sodium hydroxide; the subscripts indicate their content per 100 ml water. The amount of sodium hydroxide used was based on the amount of TA: for 1% TA, 0.587 g (the amount is calculated according to Equation (1)) sodium hydroxide is added in the solution. For the composition with sodium hydroxide without TA, the amount of sodium hydroxide was added as 0.587 wt %. All compositions are shown in Table 1. The samples were stored in a desiccator after fabrication and were dried in a vacuum oven at 50 °C before characterization.

(1) $$\;m_{NaOH}=1*\frac{25}{M_{TA}}*M_{NaOH}\;$$

### 2.3. Characterizations

The aerogel densities were calculated by measuring the mass and dimensions using a Mettler Toledo AB204-S analytical balance (Greifensee, Switzerland) and a digital caliper (Fisher Scientific, Pittsburgh, PA, USA). Every composition was tested with five samples.

The compression test was conducted on Instron model 5500 universal testing machine (Instron, Norwood, MA, USA ), fitted with a 1 KN load cell and 10 mm/min compression rate. The modulus was attained from the slope of the area under the linear portion of the stress-strain curve. Five cylindrical samples (~20 mm in diameter and height) were tested for each composition, and the results were averaged. 

Scanning electron microscopy (SEM) was conducted on a HITACHI S-4500 scanning electron microscope (Hitachi, Tokyo, Japan) at an acceleration voltage of 5 kV. Each sample was fractured in liquid nitrogen, then sputter coated with a 10 nm platinum layer on the surface for observing.

The thermal properties were measured by thermogravimetric analysis (TGA), using TGA Q550 (TA Instruments, New Castle, PA, USA). The specimens were placed on a platinum pan, equilibrated at 100 °C for 3 min, then heated to 700 °C at a speed of 10 °C/min. The process was conducted under a nitrogen flow rate of 40 mL/min.

The combustion behavior of the aerogel was measured using a cone calorimeter (Fire Testing Technology, East Grinstead, UK) in accordance with the ASTM E 1354 standard. The heat flux was set to 50 kW/m^2^. The rectangular samples (100 mm × 100 mm × 10 mm) were wrapped with aluminum foil before testing.

## 3. Results and Discussion

### 3.1. Mechanical Properties

Table 2 lists the densities and compressive moduli for the samples produced in this study. The aerogels containing sodium hydroxide and tannic acid showed shrinkage after freeze drying while maintaining their shapes in the mold. The P5 aerogel has the lowest shrinkage and the lowest solid concentration, and thus showed the lowest density. The sample densities increased with increasing solids content, as would be expected.

Due to its large number of hydroxyl groups resulting in extensive hydrogen bonding, TA would be expected to serve as a crosslinking agent in PVA aerogels [28,29,33].

**Figure 2:**
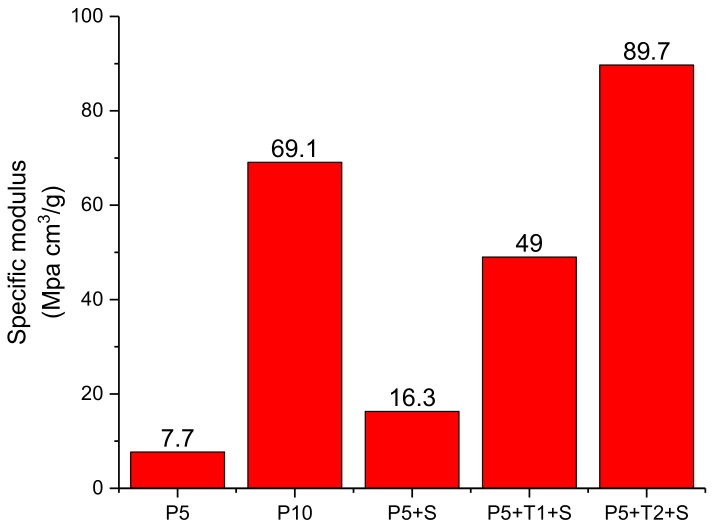
Specific moduli of aerogels with different content.

The addition of sodium hydroxide would be expected to partially deprotonate the PVA, reducing interchain hydrogen bonding, but densifying the aerogels due to higher overall levels of solids. The base would more extensively deprotonate phenolic and carboxylic acid groups on the tannic acid backbone, setting up a complex ionic structure. The base plus tannic acid combination not only increased the moduli of the PVA aerogels, but also their specific moduli (Figure 2), correcting for the higher total solids and higher bulk densities of these materials.

### 3.2. Morphology

**Figure 3:**
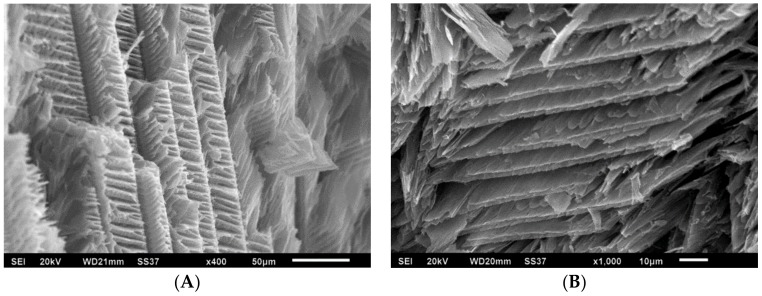

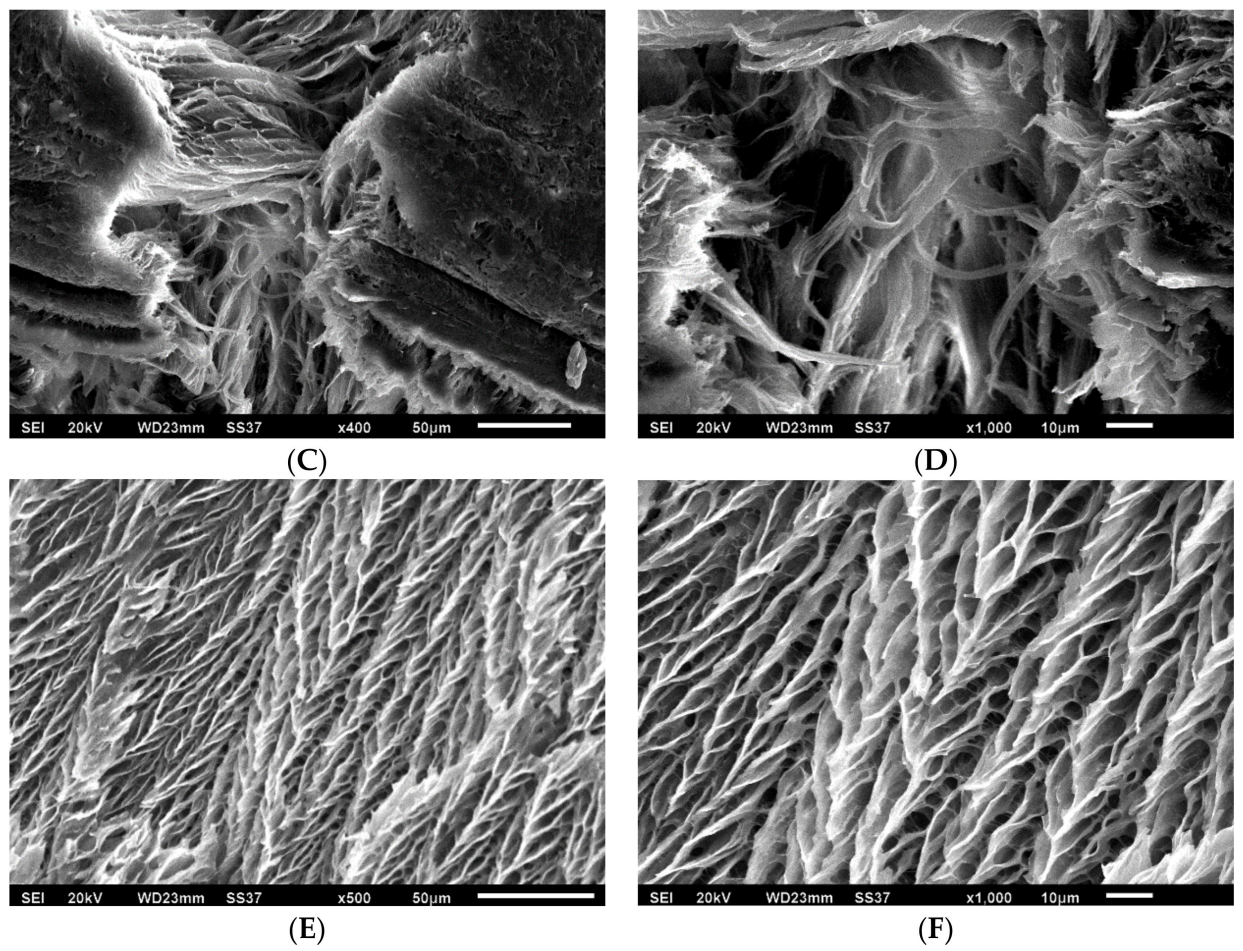
SEM images of aerogel samples. (**A**,**B**) P5; (**C**,**D**) P5/S, (**E**,**F**) P5/T2/S.

SEM was used to study the structure of aerogels and interrelationship between different compositions. As shown in Figure 3A,B, unmodified PVA aerogels exhibit lamellar (house of cards) structures typical of such materials [34]. Aerogels produced containing sodium hydroxide Figure 3C,D lack this normal structure, suggesting that interactions with the freezing water and polymer are different than in the neutral systems, disrupting the lamellar ice formation. With the addition of TA into the formulation, a more uniform, dendritic morphology was observed Figure 3E,F; the sodium hydroxide would be expected to preferentially deprotonate acidic groups on the TA, allowing for increased hydrogen bonding to the PVA, and normal hydration (and ice formation upon freezing) of the polymer/TA complex.

### 3.3. Thermal Stability

**Figure 4:**
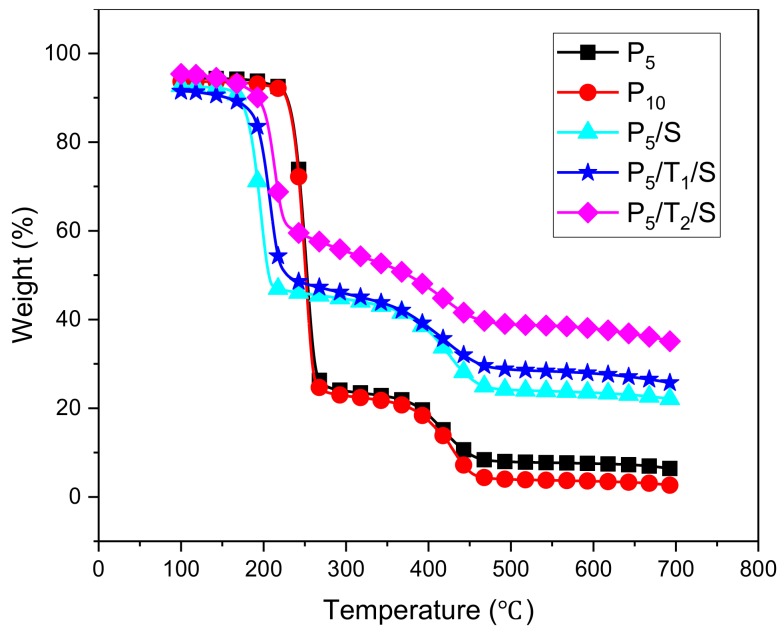
TGA curves of PVA, PVA/NaOH, PVA/TA/NaOH aerogels at a heating rate of 10 °C/min under nitrogen.

**Figure 5:**
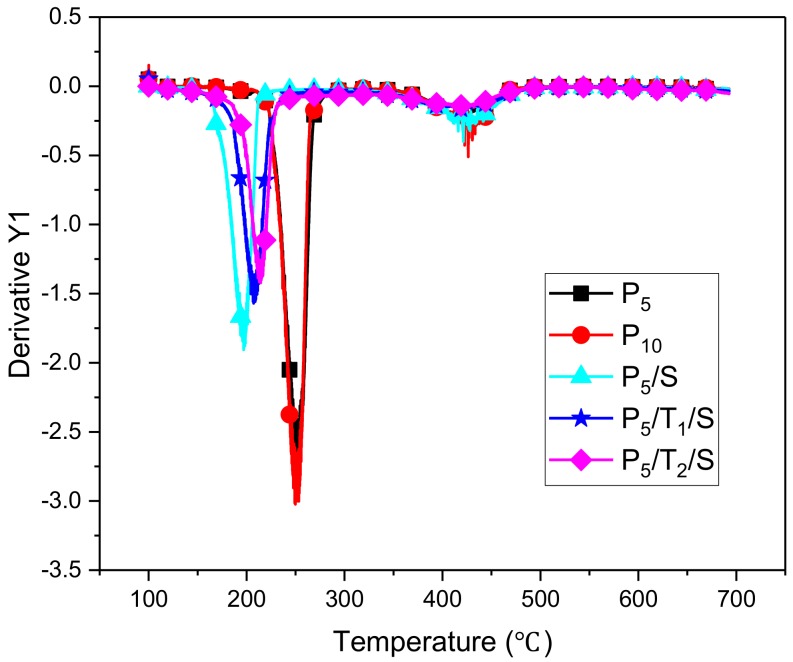
TGA curves of PVA, PVA/NaOH, PVA/TA/NaOH aerogels at a heating rate of 10 °C/min under nitrogen.

The thermal stabilities of the aerogels in this study were investigated by thermogravimetric analysis (TGA; Figure 4) and differential thermogravimetry (DTG; Figure 5). The data are summarized in Table 3, which includes the decomposition temperatures at 10% weight loss (*T*_d10%_), at 20% weight loss (*T*_d20%_), and at the maximum decomposition rates (*T*_dmax_), the values at maximum mass decomposition rate (dW/dT) and the residue. PVA aerogels are hydrophilic and could easily absorb water. To avoid the influence of moisture, the samples were heated to 100 °C at a heating rate of 40 °C/min from room temperature and then equilibrated at 100 °C for 3 min and then ramped to 700 °C at a heating rate of 10 °C/min under nitrogen.

The weight losses before 100 °C were likely the result of water desorption, a common phenomenon in hydrophilic materials. The main decomposition step occurred between 150–500 °C. The onset decomposition was evaluated by T_d10%_. With the addition of TA and NaOH, the onset temperature decreased, likely because of the effect of added sodium hydroxide as previously reported [18,32,35].

The T_d10%_, T_d20%_, T_dmax_ values of aerogels that incorporated TA and NaOH all dropped compared with the pristine PVA aerogel, while the maximum mass-decomposition rates (dW/dT) also dropped from 3.03 to 1.41 and the residue (char yield) increased from 2.6% to 35%, the corresponding sharp weight-losses are given in Figure 5. These data suggest that a char layer was formed before the bulk decomposition of the PVA matrix. This char layer could hamper the heat transfer and therefore mitigated the decomposition of PVA; this is consistent with Figure 4, which shows that the sharp weight losses were prevented by the formation of char. Both TA and NaOH exhibited positive effects on increasing the formation of char [32]. For the samples with TA, this high level of char was most likely formed from TA, a known precursor to graphite. For samples with only NaOH, the char formation argues that basic conditions/deprotonation of the polymer hinders the normal thermal degradation mechanism present with this material, allowing a carbonization pathway to occur first. Such a pathway could involve hydrogen atom transfers to produce unsaturated polymer backbones, ultimately leading to unsaturated rings. The nature of this mechanism will be the subject of a future study. With increasing TA levels, more of this known char former will be available, leading to a more robust layer, a 10% decrease of maximum mass-decomposition rates, and a 40% increase in the residue. Though the onset of the decomposition temperature dropped caused by the addition of NaOH, the addition of TA and sodium hydroxide further suppressed the weight loss during the main degradation; this effect is greatly dependent upon the concentration of TA, not NaOH, as reported [32]. The decomposition temperature was slightly enhanced by adding more TA. This may be because TA can impart better thermal stability.

### 3.4. Combustion Behavior

The combustion behavior of the aerogels was studied using cone calorimetry (Table 4); the time to ignition (TTI), peak of heat release (PHRR), time to peak of heat release (TTPHRR), the fire growth rates (FIGRA) and total heat release (THR) were compared for the samples. 

**Figure 6:**
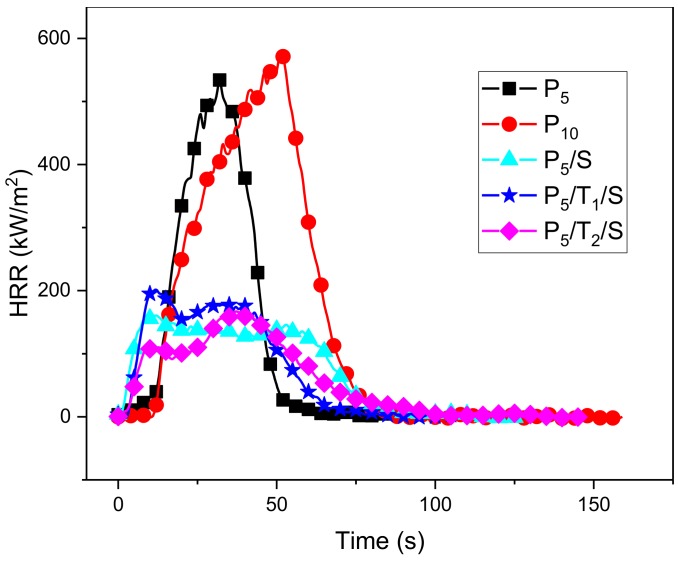
HRR plots of PVA, PVA/NaOH, PVA/TA/NaOH aerogels.

Although the samples containing TA or NaOH ignited easily under the heat flux of 50 kW/m^2^, their overall flammability was significantly reduced. The heat release rate (HRR) plot is given in Figure 6; the control PVA aerogels showed a sharp peak, while the samples containing TA showed a relatively broad doublet peak in the HRR plot. The shape of samples with TA and NaOH is a typical HRR curve of thick charring materials. The PHRR of the PVA aerogels was approximately 550 kW/m^2^; with the increase in the concentration of TA, the PHRR dropped continuously and the PHRR of P5/T2/S dropped to 166 kW/m^2^, which was about 31% of the number for pure PVA aerogel. Because of the rupture of the char during combustion, the plots of aerogels with TA showed two HRR peaks [36].

**Figure 7:**
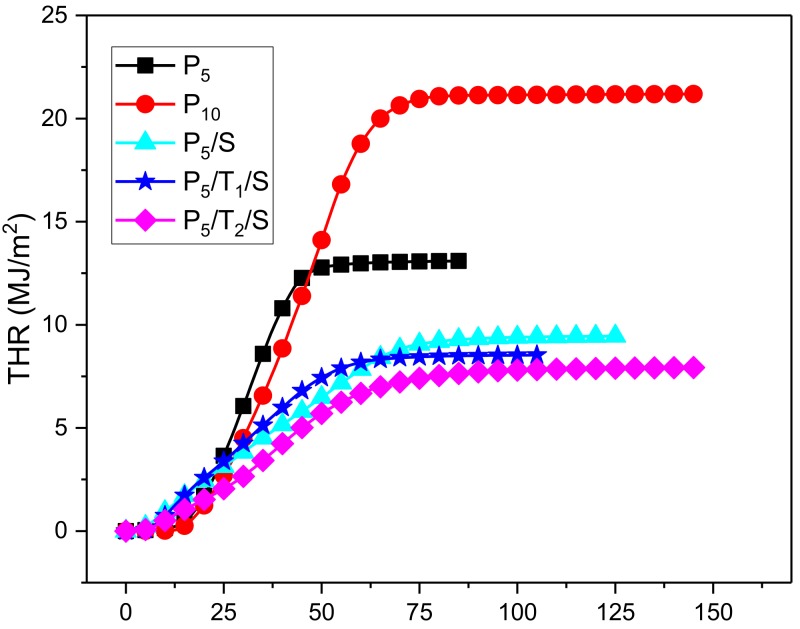
THR plots of PVA, PVA/NaOH, PVA/TA/NaOH aerogels.

Figure 7 and Table 4 presents the THR curves of the aerogels of this study. Control PVA samples exhibited a much higher THR than aerogels that incorporated TA and NaOH. To eliminate the influence of mass differences, THR/mass was compared so it could better illustrate the combustion behavior of the sample. The P5/T2/S aerogel exhibited the lowest THR/mass value, which was 1.1 MJ/(m^2^·g), 46% of the primitive PVA aerogels.

FIGRA data are given in Table 4. Lower FIGRA values indicate that the material has a lower flammability. Aerogels containing TA and NaOH showed a significant decrease in FIGRA and aerogels with higher TA concentration exhibited lower FIGRA. For the P5/T2/S aerogel, the value dropped from about 17 to 5 MJ/m^2^. The addition of large amounts of TA will decrease the fire growth rate significantly.

The data above all showed that the addition of TA and NaOH will decrease the flammability of materials. The materials containing TA and NaOH ignited earlier than pure PVA aerogels, apparently forming char rapidly, before the full matrix polymer could ignite. Aerogels with higher concentrations of TA and NaOH formed a denser char layer, which protected the matrix better from the heat and the flammable gas. TA releases 1,2,3-benzene triol and carbon dioxide as major gaseous products resulting from the decarboxylation in its outer layer of gallic acid units [26]. Sodium hydroxide could potentially facilitate decarboxylation of tannic acid at lower temperatures, which enhanced the formation of intumescent char, consistent with the flame retardancy observed with this system [32]. The aerogel modified with only NaOH also ignited rapidly, suggesting a base-catalyzed degradation of PVA which produced carbon precursors; these aerogels also formed char layer during combustion tests, consistent with their low PHRR and FIGRA compared to unmodified PVA aerogel. The char layer may form because the NaOH can produce high-carbon-content structures via deprotonation and the release of other small molecular substances during the combustion. We speculate that the deprotonated (or at least base-coordinated alcohol) may enhance beta-scission reaction, creating carbon-carbon double bonds in the polymer backbone, which ultimately could cyclize to produce aromatic char. To the best of our knowledge, this base effect on the flammability of PVA has not been reported but appears to be profound in scope. Development of a detailed mechanism for this effect will be the subject of continuing research by our team.

## 4. Conclusions

PVA aerogels with TA or TA/NaOH were prepared through a simple and environmentally friendly freeze-drying process. The densities, compressive moduli, morphologies, thermal stabilities and combustion behaviors were all investigated. By adjusting pH, homogeneous solutions of TA could be produced. Aerogels that contained TA enhanced the compressive moduli greatly, compared to control aerogels, likely due to significant hydrogen bonding between TA and PVA. The aerogels containing both NaOH and TA exhibited the highest compressive moduli, about 24 times the value of the control aerogels. SEM images showed that PVA aerogels containing TA possessed denser structures, consistent with interaction between TA and PVA. The addition of TA and NaOH decreased the onset decomposition temperature in TGA testing, but the decomposition process of PVA was hindered after the formation of significant char. Combustion tests showed that the PHRR decreased from 534 to 166 kW/m^2^and THR decreased from 12.9 to 7.5 MJ/m^2^ with the addition of TA and NaOH. The FIGRA also dropped by adding TA and NaOH. The aerogels modified only with NaOH showed a similar thermal stability and flammability to aerogels containing TA, likely due to an alternative mechanism for the production of char. The results in this study indicate that the addition of the relatively benign additives TA and NaOH can significantly decrease PVA aerogel flammability, producing a low-density, mechanically-strong material which does not require the use of halogens or phosphorous compounds in order to minimize their flammability.

## Figures and Tables

**Table 1:** Composition of aerogels.

Sample	PVA (g/%total)	Tannic acid (g/%total)	NaOH (g/%total)	DI water (g)
P5	5/100%	0/0%	0/0%	100
P10	10/100%	0/0%	0/0%	100
P5/S	5/90%	0/0%	0.587/10%	100
P5/T1/S	5/76%	1/15%	0.587/9%	100
P5/T2/S	5/61%	2/25%	1.174/14%	100

**Table 2:** Densities and compression properties of PVA aerogels, PVA/NaOH aerogel and PVA/TA/NaOH.

Sample	Density (g/cm^3^)	Modulus (MPa)	Specific modulus (MPa cm^3^/g)
P5	0.065±0.003	0.5±0.0	7.7±0.4
P10	0.124±0.002	8.6±2.3	69±18
P5/S	0.087±0.001	1.4±0.4	16±5
P5/T1/S	0.088±0.001	4.3±0.6	49±7
P5/T2/S	0.141±0.003	13±3	90±21

**Table 3:** TGA data of freeze dried PVA, PVA/NaOH, PVA/TA/NaOH aerogels.

Sample	Td10% (°C)	Td20% (°C)	Tdmax (°C)	Dw/dt (%/°C)	Weight at 100(%)	Residue (%)
P5	228	239	250	2.67	94.4	6.4
P10	228	238	250	3.03	93.6	2.6
P5/S	170	186	198	1.88	92.6	22
P5/T1/S	156	197	208	1.57	91.4	26
P5/T2/S	193	209	214	1.41	95.4	35

**Table 4:** Cone calorimeter data of PVA, PVA/NaOH, PVA/TA/NaOH aerogels.

Sample	Weight (g)	TTI (s)	PHRR (kW/m^2^)	TTPHRR (s)	FIGRA (kW/(s m^2^))	THR (MJ/m^2^)	THR/mass (MJ/(m^2^ g))
P5	5.3	8	534	32	16.7	12.9	2.4
P10	8.9	8	574	51	11.3	21.1	2.4
P5/S	5.9	3	160	12	13.4	9.4	1.6
P5/T1/S	5.8	6	201	16	12.6	8.3	1.4
P5/T2/S	6.7	7	166	37	4.5	7.5	1.1

## References

[1] Kistler S.S. (1931). Coherent Expanded Aerogels and Jellies. Nature.

[2] Bandi S., Schiraldi D.A. (2006). Glass Transition Behavior of Clay Aerogel/Poly (Vinyl Alcohol) Composites. Macromolecules.

[3] Aaltonen O., Jauhiainen O. (2009). The Preparation of Lignocellulosic Aerogels from Ionic Liquid Solutions. Carbohydr. Polym..

[4] Zou J., Liu J., Karakoti A.S., Kumar A., Joung D., Li Q., Khondaker S.I., Seal S., Zhai L. (2010). Ultralight Multiwalled Carbon Nanotube Aerogel. ACS Nano.

[5] Haq E.U., Zaidi S.F.A., Zubair M., Karim M.R.A., Padmanabhan S.K., Licciulli A. (2017). Hydrophobic Silica Aerogel Glass-Fibre Composite with Higher Strength and Thermal Insulation Based on Methyltrimethoxysilane (MTMS) Precursor. Energy Build..

[6] Gawryla M.D., Schiraldi D.A. (2009). Novel Absorbent Materials Created Via Ice Templating. Macromol. Mater. Eng..

[7] Xia W., Qu C., Liang Z., Zhao B., Dai S., Qiu B., Jiao Y., Zhang Q., Huang X., Guo W. (2017). High-Performance Energy Storage and Conversion Materials Derived from a Single Metal–organic Framework/Graphene Aerogel Composite. Nano Letters.

[8] Pajonk G.M. (1991). Aerogel Catalysts. Appl. Catal..

[9] Zhang Y., Zuo L., Huang Y., Zhang L., Lai F., Fan W., Liu T. (2015). In-Situ Growth of Few-Layered MoS2 Nanosheets on Highly Porous Carbon Aerogel as Advanced Electrocatalysts for Hydrogen Evolution Reaction. ACS Sustain. Chem. Eng..

[10] Yu Z., McInnis M., Calderon J., Seal S., Zhai L., Thomas J. (2015). Functionalized Graphene Aerogel Composites for High-Performance Asymmetric Supercapacitors. Nano Energy.

[11] Arndt E.M., Gawryla M.D., Schiraldi D.A. (2007). Elastic, Low Density Epoxy/Clay Aerogel Composites. J. Mater. Chem..

[12] Xu Z., Zhang Y., Li P., Gao C. (2012). Strong, Conductive, Lightweight, Neat Graphene Aerogel Fibers with Aligned Pores. ACS Nano.

[13] Ma C., Du B., Wang E. (2017). Self-Crosslink Method for a Straightforward Synthesis of Poly (Vinyl Alcohol)-Based Aerogel Assisted by Carbon Nanotube. Adv. Funct. Mater..

[14] Chen H., Wang Y., Schiraldi D.A. (2014). Preparation and Flammability of Poly (Vinyl Alcohol) Composite Aerogels. ACS Appl. Mater. Interfaces.

[15] Wang Y., Zhao H., Degracia K., Han L., Sun H., Sun M., Wang Y., Schiraldi D.A. (2017). Green Approach to Improving the Strength and Flame Retardancy of Poly(Vinyl Alcohol)/Clay Aerogels: Incorporating Biobased Gelatin. ACS Appl. Mater. Interfaces.

[16] Hickey A.S., Peppas N.A. (1995). Mesh Size and Diffusive Characteristics of Semicrystalline Poly (Vinyl Alcohol) Membranes Prepared by Freezing/Thawing Techniques. J. Membr. Sci..

[17] Chen H., Hollinger E., Wang Y., Schiraldi D.A. (2014). Facile Fabrication of Poly (Vinyl Alcohol) Gels and Derivative Aerogels. Polymer.

[18] Chen H., Liu B., Huang W., Wang J., Zeng G., Wu W., Schiraldi D.A. (2014). Fabrication and Properties of Irradiation-Cross-Linked Poly (Vinyl Alcohol)/Clay Aerogel Composites. ACS Appl. Mater. Interfaces.

[19] Immelman E., Bezuidenhout D., Sanderson R.D., Jacobs E.P., van Reenen A.J. (1993). Poly(Vinyl Alcohol) Gel Sub-Layers for Reverse Osmosis Membranes. III. Insolubilization by Crosslinking with Potassium Peroxydisulphate. Desalination.

[20] Trochimczuk A.W., Kabay N., Arda M., Streat M. (2004). Stabilization of Solvent Impregnated Resins (SIRs) by Coating with Water Soluble Polymers and Chemical Crosslinking. React. Funct. Polym..

[21] Liu A., Medina L., Berglund L.A. (2017). High-Strength Nanocomposite Aerogels of Ternary Composition: Poly (Vinyl Alcohol), Clay, and Cellulose Nanofibrils. ACS Appl. Mater. Interfaces.

[22] Wang L., Sanchez-Soto M., Maspoch M.L. (2013). Polymer/Clay Aerogel Composites with Flame Retardant Agents: Mechanical, Thermal and Fire Behavior. Mater. Des..

[23] Gilman J.W., Ritchie S.J., Kashiwagi T., Lomakin S.M. (1997). Fire-retardant Additives for Polymeric materials—I. Char Formation from Silica Gel–potassium Carbonate. Fire Mater..

[24] Tributsch H., Fiechter S. (2008). The Material Strategy of Fire-Resistant Tree Barks. High Performance Structures and Materials IV. WIT Trans. Built Environ..

[25] Cowan M.M. (1999). Plant Products as Antimicrobial Agents. Clin. Microbiol. Rev..

[26] Xia Z., Singh A., Kiratitanavit W., Mosurkal R., Kumar J., Nagarajan R. (2015). Unraveling the Mechanism of Thermal and Thermo-Oxidative Degradation of Tannic Acid. Thermochim. Acta.

[27] Darnerud P.O. (2003). Toxic Effects of Brominated Flame Retardants in Man and in Wildlife. Environ. Int..

[28] Chen Y., Peng L., Liu T., Wang Y., Shi S., Wang H. (2016). Poly (Vinyl Alcohol)–tannic Acid Hydrogels with Excellent Mechanical Properties and Shape Memory Behaviors. ACS Appl. Mater. Interfaces.

[29] Hong K.H. (2017). Polyvinyl Alcohol/Tannic Acid Hydrogel Prepared by a Freeze-Thawing Process for Wound Dressing Applications. Polym. Bull..

[30] Alexy P., Kachova D., Kršiak M., Bakoš D., Šimková B. (2002). Poly (Vinyl Alcohol) Stabilisation in Thermoplastic Processing. Polym. Degrad. Stab..

[31] Chu W., Yang J., Liu T., Tiu C., Guo J. (2007). The Effects of pH, Molecular Weight and Degree of Hydrolysis of Poly (Vinyl Alcohol) on Slot Die Coating of PVA Suspensions of TiO2 and SiO2. Colloids Surf. Physicochem. Eng. Aspects.

[32] Nam S., Condon B.D., Xia Z., Nagarajan R., Hinchliffe D.J., Madison C.A. (2017). Intumescent Flame-Retardant Cotton Produced by Tannic Acid and Sodium Hydroxide. J. Anal. Appl. Pyrolysis.

[33] Shi S., Peng X., Liu T., Chen Y., He C., Wang H. (2017). Facile Preparation of Hydrogen-Bonded Supramolecular Polyvinyl Alcohol-Glycerol Gels with Excellent Thermoplasticity and Mechanical Properties. Polymer.

[34] Van Olphen H. (1967). Polyelectrolyte Reinforced Aerogels of Clays---Application as Chromatographic Adsorbents. Clays Clay Miner..

[35] Nam S., Kim H.J., Condon B.D., Hinchliffe D.J., Chang S., McCarty J.C., Madison C.A. (2016). High Resistance to Thermal Decomposition in Brown Cotton is Linked to Tannins and Sodium Content. Cellulose.

[36] Schartel B., Hull T.R. (2007). Development of Fire-retarded Materials—Interpretation of Cone Calorimeter Data. Fire Mater..

